# Bone Tissue Engineering with Adipose-Derived Stem Cells in Bioactive Composites of Laser-Sintered Porous Polycaprolactone Scaffolds and Platelet-Rich Plasma

**DOI:** 10.3390/ma6114911

**Published:** 2013-10-25

**Authors:** Han-Tsung Liao, Jyh-Ping Chen, Ming-Yih Lee

**Affiliations:** 1 Department of Chemical and Materials Engineering, Chang Gung University, Kweishan, Taoyuan 333, Taiwan; E-Mail: lia01211@gmail.com; 2 Department of Plastic and Reconstructive Surgery, Chang Gung Memorial Hospital, Craniofacial Research Center, College of Medicine, Chang Gung University, Kweishan, Taoyuan 333, Taiwan; 3 Research Center for Industry of Human Ecology, Chang Gung University of Science and Technology, Kweishan, Taoyuan 333, Taiwan; 4 Department of Mechanical Engineering, Chang Gung University, Kweishan, Taoyuan 333, Taiwan; E-Mail: leemiy@mail.cgu.edu.tw

**Keywords:** bone tissue engineering, selective laser sintering, polycaprolactone, platelet-rich plasma, adipose-derived stem cells

## Abstract

Three-dimensional porous polycaprolactone (PCL) scaffolds with consistent inter-pore channels, 83% porosity and 300–400 μm pore size were fabricated via selective laser sintering. The PCL scaffold was combined with platelet-rich plasma (PRP) to form a bioactive composite and studied for potential application in bone tissue engineering using porcine adipose-derived stem cells (PASCs). The PCL/PRP/PASCs construct showed enhanced cell seeding efficiency and synergistically increased the differentiation capability of PASCs in osteogenic medium toward the osteoblast lineage, judging from elevated alkaline phosphatase activity and up-regulated osteogenic genes expression. For *in vivo* study, a 3 cm × 3 cm mandible defect was created in pigs and reconstructed by implanting acellular PCL scaffolds or PCL/PRP/PASCs constructs. Both groups showed new bone formation, however, the new bone volume was 5.1 times higher for PCL/PRP/PASCs 6 months post-operation. The bone density was less and loose in the acellular PCL group and the Young’s modulus was only 29% of normal bone. In contrast, continued and compact bone formation was found in PCL/PRP/PASCs and the Young’s modulus was 81% that of normal bone. Masson’s trichrome stain, immunohistochemical analysis of osteocalcin and collagen type I also confirmed new bone formation.

## 1. Introduction

Search for a suitable scaffold is an important and growing issue for bone tissue engineering (TE). A scaffold must be three-dimensional (3D) and have characteristics such as biodegradability, biocompatibility and pore interconnectivity [1]. Traditional methods such as particulate-leaching, phase separation, fiber bonding and freeze drying have been used to produce scaffolds for bone TE [2]. However, most of them cannot produce scaffolds with complex geometry or constant internal architecture. The pore size, porosity and even the interconnection between pores are always in random fashion within these scaffolds, which leads to specimens with great variation in microstructure from each manufacturing batch [3].

Computer-aided design (CAD) with rapid prototyping (RP) method is an advanced technique for fabricating TE scaffolds [4,5,6,7,8]. It improves the drawbacks and limitation of traditional methods. The technique possesses the advantages of fabricating scaffolds with precise control of pore shape, geometry and spatial distribution, inter-pore connection and complex 3D architecture. Several RP systems are available for producing TE scaffolds, which could be classified into three groups based on the materials deposition mechanisms [6,7]. The first group uses laser-based machines, including selective laser-sintering (SLS) and stereolithography [9]. The second group is based on material printings, including 3D printing (3-DP) and wax printing systems [10]. The third group is nozzle-based systems, including fused deposition modeling (FDM) and bio-plotters [11,12,13]. All three groups can manufacture 3D porous scaffolds with consistent microstructure for bone TE with their own advantages and disadvantages. In this study, we use SLS, a form of solid freeform fabrication to fabricate scaffolds, by selectively laser-sintering polymer powder layer-by-layer into a scaffold of desirable shape.

Different biodegradable polymers could be employed to manufacture TE scaffold by SLS subject to the limitation of thermal properties of the material. We chose poly-ε-caprolactone (PCL), a polymer suitable for SLS and potentially useful for bone TE, to fabricate scaffolds in this study [5,14,15]. PCL is a semi-crystalline polymer with good thermal stability and long *in vivo* degradation time up to two years. Its use could be translated from bench to clinical use easily due to the approval status by the U.S. Food and Drug Administration (FDA). For bone TE, scaffolds made from PCL have been shown previously to support ectopic bone formation in a rat model [16], repair orbital defects in a pig model [17], and burr hole defects in patients with chronic subdural hematoma [18].

Adipose-derived stem cells (ASCs) have been proven to be an excellent cell source for bone TE after the pioneering work by Zuk *et al.* [19,20], who reported the existence of mesenchymal stem cells in adipose tissue. ASC could be easily obtained from adipose tissue with the liposuction procedure performed by plastic surgeons under local anesthesia. Patients gain aesthetic results and suffer little discomfort from this procedure. De Ugarte *et al.* [21] demonstrated that ASCs had equal ability as bone marrow stem cells (BMSCs) to differentiate into multi-lineage cells such as osteoblasts, chondrocytes and adipocytes. Since abundant stem cells could be obtained from a single liposuction procedure, using ASCs as the cell source, TE can substantially reduce the *ex vivo* cell expansion time and avoid possible genetic mutations after long-term *in vitro* expansion. Pig adipose-derived stem cells (PASCs) isolated from pig fat tissue had also multi-lineage differentiation potentials [22].

Platelet-rich plasma (PRP) is an autogenous blood fraction with high platelet concentrations. PRP has been widely used to enhance bone formation because α-granules in PRP contain various growth factors, e.g., platelet-derived growth factor (PDGF), transforming growth factor-β (TGF-β), epidermal growth factor (EGF), insulin growth factor (IGF) and vascular endothelial growth factor (VEGF) [23,24]. Both PDGF and TGF-β are involved in connective tissue repair and bone regeneration. The α-granules are also rich in the cell adhesion molecule vitronectin, which is required for osteoconduction and osteointegration. Abundant examples in the literature have addressed the positive effect of PRP on bone regeneration by combining with different biomaterials and various cell sources. Previously, Rai *et al.* [12,25] described the combination of PRP with PCL-tricalcium phosphate (TCP) scaffold fabricated by FDM for reconstruction of rat femur nonunion defects and dog mandible defects. They found that the PCL-TCP/PRP group showed earlier neovascularization but later bone formation than the PCL-TCP group. Schuckert *et al.* [26] also successfully reconstructed the mandibular defect by combining PCL scaffolds manufactured by CAD with PRP and bone morphogenetic protein (BMP-2) in a 71-year-old female patient. We hypothesize that RP-based PCL scaffolds could become bioactive after combining with PRP to further increase the scaffold’s bone regeneration capability. Porous 3D PCL or ceramics containing PCL scaffolds fabricated via SLS have been studied previously for bone TE [27,28,29]. However, to the best of our knowledge, 3D PCL scaffolds fabricated by SLS have not been tested for enhanced osteogenic potential when combined with PRP, and there is no study addressing the osteogenic effect of seeding ASCs within the bioactive laser-sintered PCL/PRP composites.

The purposes of this paper are first to address fabricated porous 3D PCL scaffolds by the SLS technique. The laser-sintered PCL scaffold is further modified by blending it with PRP. The PCL/PRP composite is then evaluated for attachment, proliferation and osteogenic differentiation of PASCs *in vitro*. Finally, we set out to determine if the PCL/PRP composite scaffold seeded with PASCs could regenerate critical-sized pig mandible defects *in vivo*.

## 2. Results

### 2.1. Characterization of Laser-Sintered PCL Scaffold

**Figure 1:**
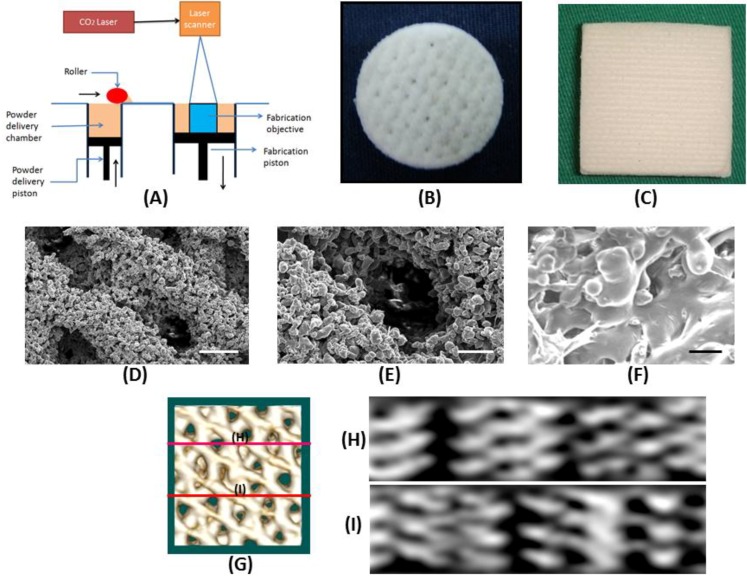
(**A**) Schematic diagram of the design of the selective laser sintering (SLS) machine, and gross views of three-dimensional porous polycaprolactone (PCL) scaffolds prepared by selective laser-sintering (SLS) for (**B**) *in vitro* and (**C**) *in vivo* studies. (**D**–**F**) Scanning electron microscope images of the microstructure of PCL scaffolds prepared by SLS. (**D**) Magnification = 45×, bar = 500 μm; (**E**) magnification = 100×, bar = 200 μm; (**F**) magnification = 250×, bar = 100 μm. (**G**–**I**) Micro-computed tomography (μ-CT) analysis of PCL scaffolds prepared by SLS. The lines in 3D image (G) mark the planes of cross-sections where 2D cross-sectional views are taken (H and I).

The house-made SLS system (Figure 1A) could produce disk-shape sintered PCL scaffolds of 10 mm in diameter and 5 mm in thickness for *in vitro* study (Figure 1B). For *in vivo* study, the PCL scaffold was sintered into a rectangular-shape with 20 layers with a size of 30 mm × 30 mm × 10 mm (Figure 1C). PCL powder could be sintered together effectively with the formation of necking between particles and a precise control of pore size and pore orientation could be achieved as observed from scanning electron microscope (SEM) (Figure 1D–F). The compressive Young’s modulus, porosity and pore size of the laser-sintered PCL scaffold are 3.75 ± 0.13 MPa, 83.1 ± 1.7% and 359 ± 43 μm, respectively. From micro-computed tomography (μ-CT) analysis, the 3D image (Figure 1G) details the geometry of the PCL scaffold while cross-section views taken at two cross-sectional planes indicate the PCL scaffold is with a porous structure containing inter-connected pores.

### 2.2. Preparation of Platelet-Rich Plasma (PRP)

The PRP is an autogenous blood fraction with high platelet concentrations, which is devoid of transmissible infectious agents and does not cause hypersensitivity reactions. The minimum platelet count to qualify as PRP is arguable, but it has been shown that a concentration of ~1 × 10^6^ platelets/μL, or approximately four to ten times more than the usual baseline platelet count in blood could produce clinical benefits [23]. To ascertain successful preparation of PRP, the mean platelet count in PRP was measured to be 2.96 × 10^6^/μL, which can be compared with the value 3.24 × 10^5^/μL in the starting blood. This indicates that the procedure could concentrate the platelets approximately nine times above the baseline platelet counts and the platelet concentration in PRP lies within the effective range mentioned above.

### 2.3. Osteogenic Differentiation of PASC in Laser-Sintered Scaffold: *In Vitro* Study

**Figure 2:**
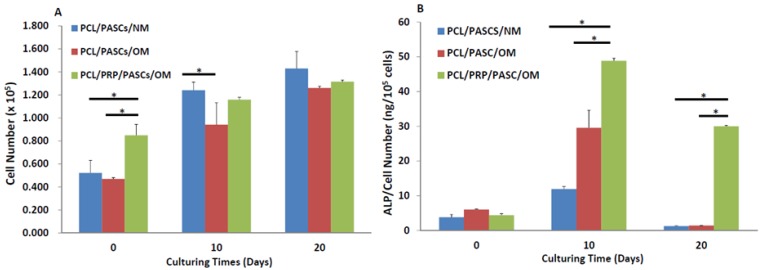
Comparison of (**A**) cell proliferation and (**B**) alkaline phosphatase (ALP) secretion of porcine adipose-derived stem cells (PASCs) in PCL and PCL/PRP scaffolds at different time points. NM, normal medium; OM, osteogenic medium. * *p* < 0.05.

As shown from Figure 2A, the cell number was significantly higher in the PCL/PRP/PASCs/OM (osteogenic medium) group than the PCL/PASCs/NM (normal medium) and the PCL/PASCs/OM group at day 0, indicating better attachment of PASCs to composite PCL/PRP scaffolds. For cell proliferation, cell number significantly increased with culturing time in each group. A significant difference in cell number was only noted at day 10 between the PCL/NM and the PCL/OM group.

The alkaline phosphatase (ALP) activity significantly elevated at day 10 and declined at day 20 during cell culture in each group (Figure 2B). The ALP activity in the PCL/PRP/PASCs/OM group is the highest at 10-day culturing time, which is 4.1 and 1.75 times that in the PCL/PASCs/NM and the PCL/PACS/OM group, respectively. At 20-day culturing time, the ALP activity in the PCL/PRP/PASCs/OM group is still the highest, and the value was raised to 23.8 and 21.3 times that in the PCL/PASCs/NM and the PCL/PACS/OM group, respectively.

**Figure 3:**
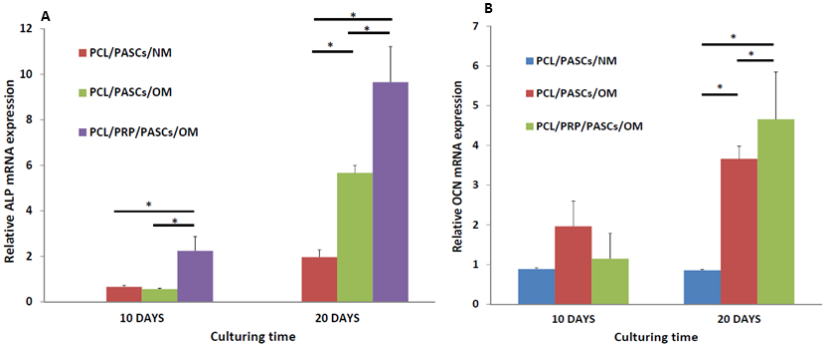
Relative (**A**) alkaline phosphatase (ALP) and (**B**) osteocalcin (OCN) mRNA expression of PASCs in PCL and PCL/PRP scaffolds at different time points. NM, normal medium; OM, osteogenic medium. The relative qRT-PCR values were corrected using the glyceraldehyde-3-phosphate dehydrogenase (GAPDH) expression levels and normalized with respect to the values on day 0 of culture. The values are the mean values ±SD of three independent experiments. * *p* < 0.05.

Comparing the specific mRNA expression of osteogenic marker genes by qRT-PCR, the ALP gene expressed early in the PCL/PRP/PASCs/OM group at day 10 and the gene expression was significantly higher (~3.7-fold) than the other two groups (Figure 3A). Nonetheless, the relative ALP mRNA expression increased and showed distinctive feature at day 20. The gene expression in the PCL/PASCs/OM and PCL/PRP/PASCs/OM group is 2.9 and 4.9-fold the value in the PCL/PASCs/NM group. Thus, augment PCL/PASCs/OM with PRP could still elevate ALP gene expression up to 70% at later times. The expression of osteocalcin (OCN) gene, a late osteogenic marker gene, was low with no significant difference among three groups at day 10 (Figure 3B). However, there was significant increase of relative OCN mRNA expression at day 20 in the PCL/PASCs/OM and the PCL/PRP/PASCs/OM group, with the levels being 4.3 and 5.5-fold the value in the PCL/PASCs/NM group. Thus, the PCL/PRP/PASCs/OM group showed 27% up-regulation of OCN gene compared with the PCL/PASCs/OM group at this time point. Taken together, those data detailed the positive effect of PRP on osteogenesis of PASCs in laser-sintered PCL scaffolds in addition to the effect of OM.

### 2.4. Bone Regeneration with Pig Critical-Sized Mandible Defect Model

**Figure 4:**
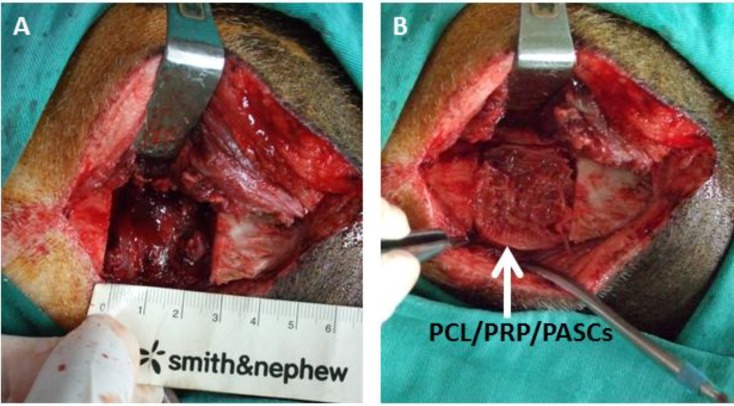
The surgery procedure for bone regeneration with a pig critical-sized mandible defect model. (**A**) A full-thickness 3cm × 3 cm bone defect was created at the lower margin of the mandibular body of a pig, which was reconstructed by (**B**) a tissue-engineered PCL/PRP/PASCs construct.

**Figure 5:**
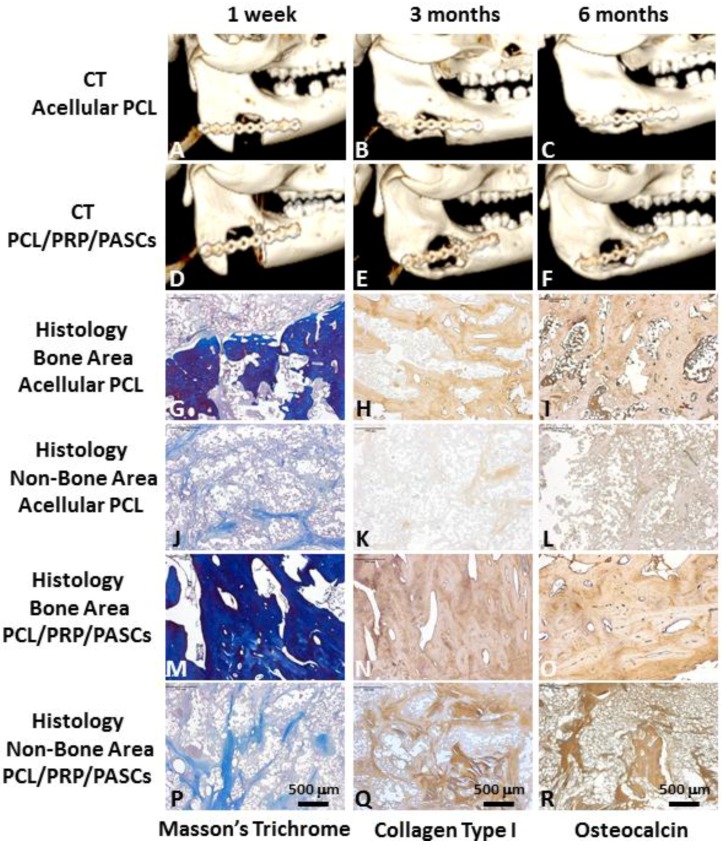
3D computerized tomography (CT) results of mandible defect reconstruction by (**A–C**) acellular PCL scaffolds and (**D–F**) PCL/PRP/PASCs constructs (**A**,**D**) 1 week, (**B**,**E**) 3 months and (**C**,**F**) 6 months post-operation. Masson’s trichrome staining, and immunohistochemical analysis of collagen type I and osteocalcin at (**G–I**) the bone formation area and (**J–L**) the non-bone formation area within the defect for mandible defect reconstruction by acellular PCL scaffolds. Masson’s trichrome staining, and immunohistochemical analysis of collagen type I and osteocalcin at (**M–O**) the bone formation area and the (**P–R**) non-bone formation area within the defect for mandible defect reconstruction by PCL/PRP/PASCs constructs.

The pigs all had uneven post-operative recovery with no wound infection, scaffold extrusion or animal death (Figure 4). Both groups demonstrated new bone formation from 3D computerized tomography (CT) analysis (Figure 5A**–**F). Loose bone formation was noted in the acellular PCL group three and six months post-operation (Figure 5B,C), indicating the 3D, porous PCL scaffold is osteoconductive. In contrast, the bone formation in the PCL/PRP/PASCs group was compact (Figure 5E,F).

The Masson’s trichrome stain showed loose bone formation within the defect in the acellular PCL group (Figure 5G). The immunohistochemistry (IHC) of collagen type I (COL I) and OCN also showed new bone formation with the positively-stained area showing the brown color (Figure 5H,I). However, both the Masson’s trichrome stain and the IHC of COL I and OCN demonstrated negative stains in the non-bone area (Figure 5J–L), which is shown as the dark area within the defect from 3D CT. For the PCL/PRP/PASCs group, the Masson’s trichrome stain showed tight and dense new bone tissue within the defect (Figure 5M). The IHC also showed more intense staining of COL I and OCN than in the acellular PCL group (Figure 5N,O). For the non-bone area within the defect in the PCL/PRP/PASCs group, we found small scattered blue bone islands from the Masson’s trichrome stain (Figure 5P). The IHC of COL I and OCN also indicated positive brown stains in the non-bone area within the defect (Figure 5Q,R). Thus, it is conceivable that active bone formation and re-modeling was progressing in the non-bone area of the PCL/PRP/PASCs construct 6 months post-operation.

**Figure 6:**
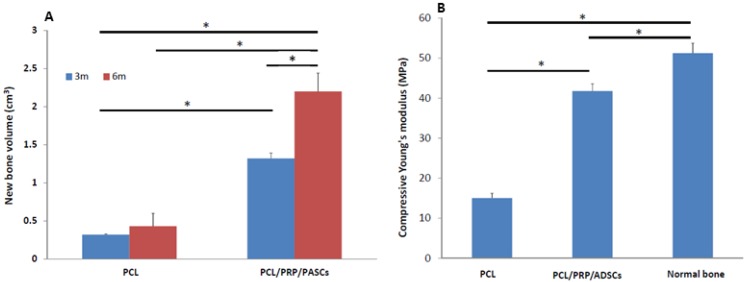
(**A**) The new bone volume calculated from 3D CT images in acellular PCL scaffolds and PCL/PRP/PASCs constructs 3 and 6 months post-operation. (**B**) The compressive Young’s modulus of acellular PCL scaffolds and PCL/PRP/PASCs contructs 6 months post-operation. * *p* < 0.05.

After calculating the new bone volume by OsiriX software (Pixmeo, Bernex, Switzerland), the acellular PCL group showed 0.32 ± 0.01 and 0.43 ± 0.07 cm^3^ new bone formation 3 and 6 months post-operation, respectively. The PCL/PRP/PASCs group regenerated 1.32 ± 0.17 and 2.20 ± 0.24 cm^3^ new bone 3 and 6 months post-operation, respectively (Figure 6A). The Young’s modulus of retrieved acellular PCL scaffold after implanting *in vivo* for 6 months was 15.05 MPa, which is 29.4% the value of normal bone (51.24 MPa) but higher than the value of the original PCL scaffold (3.75 MPa) (Figure 6B). In comparison, the Young’s modulus of the PCL/PRP/PASCs construct after implanted *in vivo* for 6 months is 41.76 MPa. Thus, the stiffness of tissue engineered bone with the PCL/PRP/PASCs construct is closer to that of normal bone at 81.6% of the modulus of normal bone.

## 3. Discussion

Bone TE has been widely studied by combining scaffolds made from different processes with various growth factors and using different cell sources. Searching for an optimal scaffold for bone TE remains an unsolved and interesting issue for researchers. The conventional techniques of porous scaffold fabrication include solvent leaching, gas foaming, vacuum drying, and phase separation. Applying those scaffolds in bone TE faces numerous challenges [3]. For example, the unequal size and uneven distribution of porogens always lead to an inconsistent pore size, and the deeply embedded porogens are difficult to be driven out for a thick scaffold. In addition, the residual organic solvents are always toxic to the human body and formation of a dense skin layer during solvent evaporation creates additional problems during cell seeding. Overall, traditional TE scaffold fabrication methods are not reproducible and cannot create a predefined and well-controlled complex micro and macro-structures.

Rapid prototyping (RP), also known as solid freeform fabrication, is an advanced method for fabricating TE scaffolds [2,4,6,30]. When used in bone TE, it can produce complex, 3D micro and macro-structure in a precise, delicate and reproducible way by CAD. Furthermore, the scaffold can be also customized to human bone anatomy into several regions and to mimic the geometry of mechanical strength by adjusting the porosities. Scaffolds created by SLS use laser energy to raise the temperature of sintered powder above its melting temperature and fuse the powder in a selective and well-defined manner. Since disadvantages of SLS may involve the difficulty of removing trapped powder and the high cost associated with commercial machines, we chose to use a SLS machine built in-house and removed trapped particles by high pressure air pumps after the SLS process. Another disadvantage of SLS is the limited choice of sintered materials. We chose PCL as the starting material in this study not only due to its low melting point for easy operation but also for its biocompatibility, biodegradability into nontoxic products, and most importantly, approval status by the U.S. FDA.

A high porosity provided by a scaffold is very important for bone TE, as it can provide enough void space for ingrowth of the surrounding bone and the vascularized tissue. Roy *et al.* [31] reported more new bone formation in areas of higher porosity in a scaffold with a porosity gradient from 80% to 88%, which was fabricated from poly(lactic-*co*-glycolic acid) and 20% TCP. A proper scaffold pore size is another important issue for bone TE. Hulbert *et al.* [32] found that the minimal pore size for regeneration of mineralized bone tissue was at 100 μm. Karageorgiou *et al.* [33] found that small pores induced osteochondral formation before osteogenesis due to relative hypoxic conditions, and large pores led to direct osteogenesis because of relatively high oxygen content by simultaneous vascularization. He concluded that a pore size larger than 300 μm is recommended for bone TE to enhance new bone and capillary formation. Oh *et al.* [34] studied the influence of pore size gradient of PCL scaffolds from 88 to 405 μm on bone regeneration with rabbit skull defect models by seeding with chondrocytes, osteoblasts and fibroblasts. The results showed a pore size ranging from 380 to 405 μm had better cell growth for chondrocytes and osteoblasts. Porous hydroxyapatite blocks of different pore sizes from 106 to 600 μm were compared for bone growth after implanting subcutaneously in rats. Higher bone formation and higher ALP and OCN content were found for blocks with 300–400 μm pore size [35]. In accordance with the results from previous studies, our PCL scaffolds fabricated by SLS could provide a suitable pore size (300–400 μm) and porosity (83%) for bone TE.

Being a relatively hydrophobic polymer with no surface signal molecules for cell recognition, PCL could not provide a good substrate for PASCs attachment. To improve this drawback, we aimed to form a bioactive composite scaffold by compounding PCL scaffolds prepared by SLS with autogenous PRP. Several advantages could be hypothesized for this modification strategy. PRP is autogenous so that there is no side effects from immune reactions after it is implanted. PRP can form platelet gel with its sol-gel transition characteristics in the presence of thrombin and calcium chloride, when fibrinogen within PRP is converted into fibrin monomer by the catalytic action of thrombin. The fibrin monomer further aggregates into a semi-solid fibrin polymer. In fact, this process is the normal blood clotting mechanism in mammalians. The sol-gel transition of PRP can help encapsulate cells within the fibrin polymer easily and evenly. Thus, in this study we first uniformly mixed PRP with PASCs in a liquid state and then induced the PRP/PASCs solution into the platelet gel/PASCs complex and simultaneously coated the complex onto the surface of sintered PCL granules within the composite scaffold.

With the α-granules within PRP contain cell-adhesive molecules (e.g., vitronectin and fibronectin), the cell seeding efficiency of PASCs to the PCL/PRP composite scaffold was enhanced 1.9-fold from the cell numbers at day 0 (Figure 2A). Although all groups showed a gradual increase of cell numbers with culturing time, the PCL/PASCs/NM group shows the highest cell proliferation rate (2.8-fold increase of cell number at day 20) followed by PCL/PASCs/OM (2.6-fold) and the lowest in PCL/PRP/PASCs/OM (1.5-fold). It is reasonable to expect that PASCs will lose their proliferation ability when induced into the osteogenic lineage. Thus, the results indicate that PRP together with OM showed a synergistic effect on osteogenic differentiation of PASCs and is accompanied by a loss of cell proliferation ability.

Successful osteogenesis of stem cells should be verified by biochemical analysis of specific biomarker proteins and gene expression analysis of osteogenic marker genes at different stages. Usually, high level expression of COL I and ALP were found in the early stage. ALP was believed to be an earlier marker before matrix maturation [36]. It is abruptly elevated at the early osteogenic period and declines gradually at later stages. ECM maturation is followed in the second stage after the high expression of ALP and finally the ECM is mineralized at the last stage. As one of the most abundant proteins in bone tissue second only to COL I, OCN is a specific marker for bone formation in the late stage, which is significantly up-regulated during matrix formation and mineralization [36]. In our study, the ALP activity was abruptly elevated at day 10 for both OM groups with the value in the PCL/PRP/PASC group higher than that in the PCL/PASC group (Figure 2B). Although the ALP activity reduced at day 20, the PCL/PRP/PASC/OM group still showed dramatic enhancement of ALP activity (more than 20 times higher) compared with other groups, providing direct evidence for enhanced osteogenesis of PASCs in the presence of PRP. The enhanced expression of the early osteogenic marker gene (ALP) at both day 10 and 20, and the late osteogenic marker gene (OCN) at day 20 in the PCL/PRP/PASC/OM group further supports this beneficial effect from PRP (Figure 3). Comparing the results in NM and OM also indicates that PRP works synergistically with OM to up-regulate osteogenic gene expression. Indeed, taken together the gene expression and biochemical analysis results, the PCL/PRP composite scaffold provides the best environment for osteogenic differentiation of PASCs *in vitro*.

However, good results from bench studies could not guarantee similar bone TE strategy works for animals or human because the *in vivo* environment is more complicated and complex than the *in vitro* culture condition. Thus, an animal model is required for proof of our bench results. For future clinical translation, we chose pigs as our animal model due to its similar anatomic structure and physiologic status to human. The 3D CT image showed new bone formation in both the acellular PCL and the PCL/PRP/PASCs groups. However, the newly formed bone in acellular PCL was loose and discontinuous within the defect. In contrast, the new bone was compact and bridged between the two ends of defect in the PCL/PRP/PASCs group (Figure 4). The new bone volume in the PCL/PRP/PASCs group was also 5.1 times higher 6 months post-operation (Figure 5A). The Manson’s trichrome staining and IHC of COL I and OCN further confirmed the high density area found in 3D CT was bone tissue. Although there was still defect remaining in the PCL/PRP/PASCs group, the histology and IHC results indicated active osteogenesis in the defect area due to the positive stains of COL I and OCN in the non-bone area (Figure 4P–R). The strength of tissue engineered bone is an important issue especially in the load-bearing mandible. Inadequate bone strength will result in unpredictable fracture while the mandible was functioning at mastication. The mechanical strength of laser-sintered PCL scaffold is only 3.94 MPa, which is around 7.6% of normal bone. However, after implanting into the mandibular defect, the stiffness of PCL was elevated to around 29% of normal bone (Figure 5B). Undoubtedly, the increased stiffness was due to filling of pore structure with fibrotic tissue and bone ingrowth (Figure 4G–I). Since the ingrowth of bone tissue was loose, the stiffness only increased 3.8 times after implanting acellular PCL. However, after adding PRP that contains multiple growth factors and osteo-induced PASCs into the PCL scaffold, the mechanical strength of the PCL/PRP/PASCs construct increased dramatically to 81% of the value in normal bone. A possible explanation for the inferior stiffness even in the PRP/PASCs/PCL group when compared to normal bone may be due to the limited time frame of new bone formation, where immature woven bone was formed instead of the remodeled mature bone. Also, as new bone formation was not complete throughout the scaffold, the remaining unabsorbed PCL also weakened the new bone structure. Nonetheless, from the outcomes of animal studies, our results demonstrated that laser-sintered PCL scaffold is endowed with the osteoconductive property due to the macroporous structure and the inter-connected pores within the scaffold, which could guide the ingrowth of surrounding bone tissue to form new bone within. Most importantly, the PCL/PRP/PASCs construct not only preserved the osteoconductive property of PCL scaffold but also further conferred it with osteoinductive potential, originating from PRP and the osteogenesis capability of osteo-induced PASCs.

## 4. Experimental Section

### 4.1. Adipose-Derived Stem Cells Harvest and Isolation

Animal experiments were performed with approval from the Institutional Animal Care and Use Committee of Chang Gung Memorial Hospital and conformed to the standards of the Association for Assessment and Accreditation of Laboratory Animal Care. PASCs were harvested and isolated according to our previous report [37]. Briefly, the fat tissue was harvested from the inguinal area of the pig after sterilization of the lower abdomen with beta-iodine solution. The fat was diced into small pieces by scissors in sterile procedure. The diced fat tissue sample was washed extensively with phosphate-buffered saline (PBS), and the extracellular matrix (ECM) was digested with 0.05% collagenase in a 37 °C water bath shaker at 165 rpm for 30 min. Enzyme activity was neutralized by adding an equal volume of normal medium (NM) containing Dulbecco’s Modified Eagle Medium (DMEM) and 10% fetal bovine serum (FBS), and centrifuged at 250 *g* for 10 min to obtain a high density cell pellet. After removing the supernatant, the cell pellet was re-suspended in 160 mM NH_4_Cl and incubated at room temperature for 10 min to lyse the contaminated red blood cells. The cell pellet was recollected by centrifugation as described above, filtered through a 100 μm nylon mesh to remove cellular debris and incubated overnight at 37 °C in a 5% CO_2_ environment in NM in culture dishes. Following incubation, the culture dishes were washed extensively with PBS to remove residual non-adherent red blood cells and the PASCs-enriched density fraction was collected. To induce osteogenic differentiation, osteogenic medium (OM) containing osteogenic supplements (DMEM, 10% FBS, 100 nmol/L dexamethasone, 50 μg/mL ascorbic acid and 10 mmol/L glycerophosphate) was used.

### 4.2. Porous Scaffold Design and Fabrication

The SLS procedure begins with a three-dimensional (3D) CAD file that is oriented in an appropriate orientation for fabricating the desired scaffold. The file is mathematically processed into two-dimensional (2D) cross sections (layers) along the Z-axis. The SLS system was house-made and consisted of a CO_2_ laser, a scanner, a platform with pre-heating system, a building magazine and a computer to input the 3D CAD file (Figure 1A). The build chamber was heated to a temperature just below the melting point of PCL powder so that heat from the laser only needed to elevate the temperature slightly to cause sintering. After filling the PCL powder into the build chamber evenly, a CO_2_ laser beam steered by a scanning system drew the 2D cross section on the surface of the build material, sintering the PCL powder. The piston of build chamber then descended a layer thickness. The PCL powder again was filled into the build magazine, where the next cross section was sintered to the previous one. This procedure was repeated until obtaining the finished scaffolds. The direction of laser sintering was designed to be 0°/45°/90°/135° with respect to the X-axis for each layer in sequential order. The PCL powder used was in a particular form with a melting point of 60 °C, a molecular weight of 50,000 Da and a particle size distribution from 30 to 50 μm. Processing of the PCL powder was conducted by preheating the powder to 40 °C and scanning the laser (450 μm focused beam diameter) at 1 W power and 500 μm/s scanning speed.

### 4.3. Characterization of Laser-Sintered PCL Scaffold

The laser-sintered PCL scaffold was analyzed for its compressive modulus, porosity and pore size. The compressive Young’s modulus was measured by a hydraulic MTS 810 material testing system (MTS Systems Corporation, Eden Prairie, MN, USA) and compressed with a crosshead speed of 1.0 mm/min. Each sample was compressed to approximately 10% strain and then released at the same rate. The compressive modulus was measured as the slope within the linear elastic region of deformation and repeated for three samples per group. For pore size measurement, scaffold samples were coated with gold/palladium (40/60) and examined using a scanning electron microscope (SEM, S3000N, Hitachi, Tokyo, Japan) at 15 kV. The mean pore size was determined by Image-J software from ten areas within each specimen. Porosity of the scaffold was measured by a liquid displacement method with ethanol as the displacement liquid. The PCL scaffold was analyzed by micro-computed tomography (μ-CT; BioScan, Paris, France) for further viewing the geometry and inter-pore connection. The helical CT data were acquired using a high-resolution frame as a set-up in the system, with tube voltage 55 KeV, pitch 1.0 and projection 180. The axial scanning range was set at 3 cm, with the PCL scaffold at the centre of the field of view. CT images with a matrix size of 280 × 280 × 300 pixels and an isotropic voxel size of 0.1 mm were reconstructed. The 3D surface-rendering images set at soft tissue-skin mode were generated by OsiriX software to detail the geometry of the PCL scaffold and the 2D cross-sectional view to confirm the pore structure and the inter-pore connection within the scaffold.

### 4.4. Preparation of Platelet-Rich Plasma (PRP)

About 250 mL of whole blood was drawn from a pig’s femoral artery and placed in a bag containing citrate phosphate dextrose solution (2.51 g/L sodium dehydrogenate phosphate, 26.30 g/L sodium citrate, 3.27 g/L citric acid and 23.30 g/L glucose) as an anticoagulant. The PRP was obtained by double-spin centrifugation [38]. Briefly, the whole blood was poured into 50 mL tubes and centrifuged at 400 *g* for 10 min. The first centrifugation step separated the platelet layer from the plasma and red blood cells. The lower red blood cells layer was discarded and the upper plasma layer and the middle platelets layer were collected and centrifuged again at 800 *g* for 10 min. The precipitated platelets were collected with part of the plasma as the PRP. The platelet count of the whole blood and PRP was carried out by a CELL-DYN Emerald hematology analyzer (Abbott, Abbott Park, IL, USA).

### 4.5. Osteogenic Differentiation of PASCs in Laser-Sintered PCL Scaffold: *In Vitro* Study

Second passage PASCs were used for *in vitro* studies of osteogenic differentiation. A disk-shape PCL scaffold (5 mm in thickness and 10 mm in diameter) was sterilized by 75% alcohol followed by UV irradiation for one day and washed with sterilized PBS several times to remove residual alcohol before cell seeding. The experiments were divided into three groups. Group I (PCL/PASCs/NM): PASCs (~1 × 10^5^ cells) were seeded in PCL scaffolds and cultured in NM. Group II (PCL/PASCs/OM): PASCs (~1 × 10^5^ cells) were seeded in PCL scaffolds and cultured in OM. Group III (PCL/PRP/PASCs/OM): PASCs (~1 × 10^5^) were mixed with PRP, followed by loading PCL scaffolds with PRP/PASCs in the presence of 100 U bovine thrombin and 10% calcium chloride to form PRP/PCL/PASCs constructs and cultured in OM. The functions of thrombin and calcium chloride were to initiate the coagulation mechanism and release growth factors from α-granules of platelets. The cell proliferation, alkaline phosphatase (ALP) activity and osteogenic mRNA expression of osteocalcin (OCN) and ALP were compared among three groups at day 0, 10 and 20. Day 0 represented 6 h after cell seeding on the scaffold. There were six samples in each experiment.

#### 4.5.1. Cell Proliferation

The proliferation of PASCs was determined by CellTiter 96 AQueous One Solution Reagent (Promega Co., Madison, WI, USA), which contains a novel tetrazolium salt (MTS). The MTS tetrazolium compound is reduced by living cells into a colored formazan product that is soluble in tissue culture medium. The quantity of formazan product is directly proportional to the number of viable cells. MTS assays were performed by adding 20 μL of MTS solution to each specimen in 100 μL culture medium and incubating at room temperature for 3 h with protection from light. Colorimetric measurement of the formazan dye was performed at a wavelength of 492 nm using an ELISA plate reader (Molecular Devices, Sunnyvale, CA, USA). Cell numbers were determined using a calibration curve relating the number of PASCs and the absorbance value.

#### 4.5.2. Alkaline Phosphatase (ALP) Activity

To analyze ALP activity, cell/scaffold constructs were washed with PBS three times and then lysed with sonication. Each specimen were suspended in 500 μL of 0.1% Triton X-100 detergent with 5 mM MgCl_2_, centrifuged at 13,000 rpm for 10 min at 4 °C, and the supernatant was assayed for ALP activity. The ALP activity was determined by measuring the release of *p-*nitrophenol from *p-*nitrophenyl phosphate (Sigma-Aldrich, St Louis, Mo, USA). Briefly, 80 μL of the supernatant was added to 100 μL of 5 mM *p*-nitrophenyl phosphate in 150 mM 2-amino-2-methyl-1-propanol buffer solution and incubated for 15–45 min at 37 °C before adding 50 μL 0.2 N NaOH to stop the enzymatic reaction. The absorbance values were determined at 405 nm, converted to concentration values with a standard curve and normalized to cell numbers for all specimens.

#### 4.5.3. Expression of Osteogenic Marker Genes by Quantitative Real-Time PCR (qRT-PCR)

Total RNA of each specimen was isolated with Trizol reagent (Invitrogen, Carlsbad, CA, USA) following the manufacturer’s protocols. Isolated RNA was dissolved in RNase-free water and the amount of RNA was determined by measuring the absorbance value (OD) at 260 nm with a spectrophotometer. RNA quality was verified by measurement of OD_260_/OD_280_. The cDNA was prepared from 2 μg of total RNA with RevertAid First Strand cDNA Synthesis Kit (Thermo Scientific Molecular Biology) in a final volume of 20 μL. Specific osteogenic marker genes, ALP and osteocalcin (OCN), were tested for osteogenesis. The primers sequence for ALP (forward primer: 5’-ATGAGCTCAACCGGAACAA-3’, reverse primer: 3’-GTGCCCATGGTCAATCCT-5’) and OCN (forward primer: 5’-TCAACCCCGACTGCGACGAG-3’; reverse primer: 3’-TTGGAGCAGCTGGGATGATGG-5’) were designed using the Oligo 6.0 program (Molecular Biology Insights, Inc., Cascade, CO, USA). For a single PCR reaction amounting to 20 μL, 0.2 μL of cDNA was used. To make the visualization of PCR products possible in real time, a SYBR Green I supermix (Yeastern Biotech Co., Taipei, Taiwan) was used. A three-temperature cycling, consisting of a denaturation step at 95 °C for 30 s and annealing step at 57.6 °C for 30 s and extension step at 72 °C for 30 s, was carried out in an iCycler iQ5 real-time detection system (Bio-Rad Laboratories Inc., Hercules, CA, USA). The specificity of each PCR reaction was assessed by performing melting curve analysis after each reaction. Glyceraldehyde-3-phosphate dehydrogenase (GAPDH) acted as a housekeeping control. Results were quantified for osteogenic marker genes using the 2^−ΔΔ*Ct*^ relative quantification method with respect to the control time point at day 0. The expression of each gene was evaluated in triplicate.

### 4.6. Bone Regeneration with Pig Critical-Sized Mandible Defect Model

Surgery was performed under sterile conditions by using endotracheal isoflurane anesthesia after induction with intravenous 4% sodium pentobarbital (1 mL/10 kg). The surgical area of the mandible in each pig was shaved before the procedure, and the surgical field was prepared with betadine solution. The mandibular angle and body were exposed through a submandibular incision. A full-thickness bone defect, 3 cm × 3 cm, was created bilaterally at the lower margin of the mandibular body by using a reciprocating saw under extensive cooling with saline. The *in vivo* experiments were divided into two groups. Group I (PCL): the defect was filled with a rectangular acellular PCL scaffold (30 mm × 30 mm × 10 mm). Group II (PCL/PRP/PASCs): the defect was filled with a PRP/PCL/PASCs construct. The construct was prepared by combining the rectangular PCL scaffold (30 mm × 30 mm × 10 mm) with PRP/PASCs (~1 × 10^8^ cells) in the presence of 100 U bovine thrombin and 10% calcium chloride and cultured in OM for 2 weeks. After achieving hemostasis, the flap was re-sutured with 4–0 nylon (Ethicon) to completely cover the wound area.

#### 4.6.1. Computerized Tomography (CT) Analysis

All animals underwent postoperative CT examination 1 week, 3 months, and 6 months post-operation. The CT image acquisition, processing, and manipulation were performed according to the standard protocol at a medical facility. The CT data were reformatted, and a voxel (unit of 3D image) was set at 0.6 mm × 0.6 mm × 0.6 mm for all scans. The imaging data were analyzed using the OsiriX Image software (version 3.6.1) for extraction and volumetric measurement of the new bone growth in the mandibular defect. The craniofacial bone was extracted from the 3D CT images with the threshold adjusted to remove the soft tissue and display the bone density. The range of CT densities was fixed in all CT scans for craniofacial bone. The new bone formation volume was calculated by region of interest (ROI) manager built in the OsiriX Image software (Pixmeo, Bernex, Switzerland).

#### 4.6.2. Histological Analysis

Specimens from the bone defect area were harvested for each group 6 months post-operation. Samples examined by histology were fixed in 4% paraformaldehyde solution, dehydrated, and embedded in paraffin. Serial sections (4 μm thick) were subject to Masson’s trichrome staining and immunohistochemical analysis. For immunohistochemical analysis, the deparaffinized sections were washed three times in Tris-buffered saline (TBS) for 5 min. Nonspecific binding sites were blocked with Dako Antibody Diluent with Background Reducing Components (Dako, Carpinteria, CA, USA) for 15 min, and then incubated overnight at 4 °C with mouse anti-collagen type I antibody (Abcam, Cambridge, UK) at 1:200 or mouse anti-osteocalcin antibody (Abcam) at 1:100. After rinsing in TBS, the sections were incubated in universal biotinylated secondary antibody in a sealed immunochamber for 12 min. Then the sealed immunochamber was covered with drops of streptavidin conjugated to horseradish peroxidase for 25 min. Peroxidase activity was visualized using 3-3’-diaminobenzidine (DAB) as the substrate. The sections were incubated with 0.06% DAB in 0.1 M Tris-HCl (pH 7.5) containing 0.03% H_2_O_2_. The sections were then counter-stained with hematoxylin for 5 min. All incubations were conducted in a humidified chamber.

#### 4.6.3. Biomechanical Testing

The compressive modulus of the newly formed bone was tested at 37 °C with a hydraulic MTS 810 material testing system (MTS Systems Corporation, Eden Prairie, MN, USA) and compressed with a crosshead speed of 1.0 mm/min. Each sample was compressed to approximately 10% strain and then released at the same rate. The compressive modulus was measured as the slope of the linear elastic region of the deformation and repeated three times per specimen.

### 4.7. Statistics

All of the data are reported as mean ± standard deviation. Statistical analyses among the multiple group data were carried out using a one-way analysis of variance (ANOVA) test to determine the significant differences. Turkey’s *post-hoc* test was used to determine the difference between any two groups with *p* < 0.05 considered statistically significant.

## 5. Conclusions

Our study demonstrates SLS could successfully fabricate 3D porous PCL scaffolds with consistent inter-pore channels, 83% porosity and 300–400 μm pore size. Combining laser-sintered PCL scaffold with PRP leads to a bioactive composite scaffold with enhanced cell seeding efficiency. The PCL/PRP/PASCs construct synergistically increases the differentiation capability of PASCs in OM toward the osteogenic lineage, as judged from elevated ALP activity and up-regulated osteogenic genes expression. The animal study indicates the PCL/PRP/PASCs construct could augment the osteoconductive property of laser-sintered PCL scaffold with the osteoinductive differentiation of PASCs in the presence of PRP, judging from more compact bone formation and improved mechanical strength of the tissue engineered bone.
